# Personal and Clinical Predictors of Poor Metabolic Control in Children with Type 1 Diabetes in Jordan

**DOI:** 10.1155/2019/4039792

**Published:** 2019-07-04

**Authors:** Abeer Alassaf, Rasha Odeh, Lubna Gharaibeh, Sarah Ibrahim, Kamel Ajlouni

**Affiliations:** ^1^Department of Pediatrics, The University of Jordan, Amman, Jordan; ^2^Department of Clinical Pharmacy, The University of Jordan, Amman, Jordan; ^3^The National Center (Institute) for Diabetes, Endocrinology and Genetics (NCDEG), The University of Jordan, Amman, Jordan

## Abstract

**Background:**

Achieving adequate metabolic control in children with type 1 diabetes is important in slowing the progression of future microvascular and macrovascular complications, but still it is a universal challenge. We aim to investigate possible factors associated with poor metabolic outcomes in Jordan as an example of a country with limited resources.

**Methods:**

This is a retrospective chart review study of children with type 1 diabetes. Several clinical and personal characteristics were tested for association with metabolic control reflected by HbA1c levels. Linear logistic regression analysis was used to evaluate possible predictors of metabolic control. One-way ANOVA analysis was used to detect significant differences in HbA1c between categories.

**Results:**

Significant predictors of metabolic control were found. A one-year increase in age led to an increase in HbA1c by 0.053% (*P* = 0.044). A decline in HbA1c levels was predicted in children who have precise amount of carbohydrates or who are receiving insulin at school (-0.46% (*P* = 0.014) and -0.82% (*P* = 0.004), respectively). When family members other than mothers decided the insulin dose, the HbA1c level increased by 0.74% (*P* = 0.005).

**Conclusion:**

Poor metabolic control was associated with age, dietary noncompliance, not receiving insulin at school, and absence of direct mother care. Our study is one of the few studies from Middle East evaluating predictors of metabolic control. Global research studies help in giving universal insight towards developing more effective multidisciplinary team approach for diabetes care and education.

## 1. Introduction

The Diabetes Control and Complications Trial (DCCT) and the Epidemiology of Diabetes Interventions and Complications (EDIC) study showed that the progression of microvascular complications including retinopathy, nephropathy, and neuropathy can be reduced by strict glycemic control [1]. HbA1c levels less than 7.5% (58.5 mmol/mol) are recommended to reduce future complications according to the International Society for Pediatric and Adolescent Diabetes (ISPAD) Clinical Practice Consensus Guidelines in 2014 [2]. Achieving adequate metabolic control requires a multidisciplinary team approach that involves the parents and their children [3, 4]. Despite these recommendations, several studies showed that most of children and adolescents with type 1 diabetes did not achieve target HbA1c levels of less than 7.5% [5–7]. Keeping blood glucose levels under control becomes more difficult as children grow older due to the influence of pubertal hormones and the decline of self-care practices [8]. Possible predictors of glycemic control were previously studied, including demographic factors such as age [9], age at diagnosis [10], and socioeconomic status [11], to name a few. Diabetes-related characteristics were also evaluated and found to be associated with metabolic control, including disease duration [12], parental attitude and involvement [13], and frequency of blood glucose self-monitoring [14]. In order to reduce adulthood diabetes-related complications, it is important to identify children with poor metabolic control risk factors, address modifiable factors, specially those related to diabetes care, and focus on children with nonmodifiable risk factors. The paucity of studies that evaluate predictors of metabolic control from the developing countries [6, 15–18], the cultural differences in terms of beliefs and preferences, and the availability of healthcare resources probably influence the metabolic control of diabetes and collectively underscore the importance of performing such studies in individual populations in order to develop strategies for improvement. The aim of this study is to identify possible personal and clinical predictors of glycemic control in children with type 1 diabetes in the understudied Jordanian population and to examine differences in glycemic control expressed as HbA1c levels between different groups.

## 2. Methods

The present study is a descriptive and retrospective cohort study of children with T1DM followed at the pediatric endocrine clinics in two institutions: Jordan University Hospital (JUH) and the National Centre for Diabetes, Endocrinology and Genetics (NCDEG). An ethical approval was obtained from the Institutional Review Boards (IRB) of both institutions and was conducted in accordance with the Declaration of Helsinki.

Data were collected by reviewing medical records using a chart checklist form. Patients were eligible for the study if they were younger than 18 years of age and had type 1 diabetes with at least one year of follow-up. Two hundred and seventy-four medical records were reviewed; eleven cases were excluded due to relevant data being missing, and thus, the final count of participants became 263. For each participant, the mean HbA1c from the most recent year of follow-up was categorized into <7.5%, 7.5-9%, and >9%, reflecting optimal, suboptimal, and poor metabolic control, respectively (ISPAD Clinical Practice Consensus Guidelines 2014) [2]. HbA1c was measured by the Bio-Rad D-10™ Dual Program using ion-exchange high-performance liquid chromatography, with a normal reference range of 4.2-6.2%.

Several independent demographic and clinical factors were analyzed as possible predictors of glycemic control. For the comparison, the age at acquisition of data and duration of diabetes were classified into groups aligned to other studies [10, 19, 20]. Age at diagnosis, gender, calculated body mass index (BMI) SDS in the latest clinic visit, and presence of any comorbidity (including autoimmune thyroiditis and celiac disease) were included in the study. Diabetes care-related factors were also analyzed including dietary compliance as reflected by counting carbohydrates, whether insulin is taken at school, who is the caregiver deciding on the insulin doses, frequency of self-monitoring blood glucose (SMBG), the number of diabetes clinic visits during the latest year of study, and the insulin regimen during the last year of follow-up. A change in insulin regimen—at any time after the diagnosis of diabetes—from two or three injections of insulin per day to a basal-bolus injection regimen or from any injection regimen to an insulin pump, was considered an upgrade. Academic achievement was assessed in terms of school grades rated out of 100.

### 2.1. Statistical Analysis

Statistical analysis was performed using IBM SPSS Statistics for Windows, version 23 (IBM Corp., Armonk, N.Y., USA). Categorical data were compared using Pearson Chi-Square and Fisher's exact test. The comparison of continuous variables among groups was conducted using one-way ANOVA. The Scheffe post hoc analysis was used to identify the groups that were significantly different from each other. Possible predictors of poor metabolic control were analyzed using linear logistic regression (backward method). *P* values less than 0.05 were considered statistically significant.

## 3. Results

The characteristics of the 263 participants who fulfilled the inclusion criteria are shown in Table 1. The mean age of participants was 11.1 ± 3.7 years, with a mean duration of diabetes of 3.8 ± 2.4 years. The mean HbA1c was 8.7% ± 1.5. Fifty-five patients (20.9%) achieved adequate metabolic control (HbA1c < 7.5%).

Differences in HbA1c levels were evaluated among various subgroups (Table 2). Post hoc analysis was used to determine significant differences between different categories. Older children (10-15 years, ≥15 years) had significantly higher HbA1c values than younger children between 5 and 10 years old (*P* = 0.007 and 0.015, respectively). Children with higher school grades (90-100) had significantly lower HbA1c values than those with lower grades (<80 and 80-90) with *P* values = 0.026 and 0.027, respectively. Glycemic control is better when the dose of insulin is determined either by the mother or by the diabetic child; HbA1c levels were significantly lower in the group of children under their mothers' direct care compared to those who have caregivers other than their mothers deciding their insulin doses. Children who do not receive insulin at school have significantly higher HbA1c values than those who do (*P* value = 0.020).

The linear regression analysis model used to predict metabolic control (Table 3) revealed that current age, dietary compliance reflected by counting carbohydrates, taking insulin injections at school, academic achievement, and the person who decides the insulin dose were predictors of HbA1c levels. Every one-year increase in age resulted in a 0.065% increase in HbA1c. Counting carbohydrates led to a decline in HbA1c by 0.503%, and receiving insulin at school led to a decrease in HbA1c by 0.743%. The HbA1c levels of children who had their insulin doses determined by caregivers other than their mothers were significantly increased by 0.58%. Children with high school grades had a decrease in HbA1c by 0.353% compared to children with low school marks; fifty percent of these children were 10 years of age or older.

## 4. Discussion

Overall, our study shows that optimal metabolic control as reflected by HbA1c < 7.5% is achieved in 20.9% of patients, which is similar to results from other studies done in developed countries [21, 22]. Gender is not associated with the metabolic control similar to what was indicated by a meta-analytic study [23]. Age correlates with HbA1c levels, starting from age group 5-10 years, as age increases so does the HbA1c. The oldest age group has a significant reduction in glycemic control which can be explained by hormonal changes and decline in parental supervision over different clinical aspects of diabetes care in the adolescents [24]. Similar to other reports, the longer the duration of diabetes, the lower the metabolic control becomes [19]. In this study, age at diagnosis is not associated with HbA1c levels and thus is not a predictor of metabolic control, unlike a previous study where older age at diagnosis was a risk factor for reduced glycemic control [10]. The frequency of celiac disease is lower than in a study conducted in Saudi Arabia [16]. Being overweight is a significant predictor of poor glycemic control. The correlation between BMI and metabolic control was controversial in several studies [13, 25]. Despite the difficulty in determining causality due to the retrospective nature of our study, we anticipate that dietary noncompliance is a major factor. Patients with higher school grades have significantly lower HbA1c values than those with lower grades. High academic achievement may reflect the individual's competency in dealing with diabetes care and hence better metabolic control [26]. On the other hand, having adequate metabolic control with less blood glucose fluctuation may reflect better school performance [27].

Frequent clinic visits in our study are associated with poor metabolic control which may be explained by the need of closer monitoring and follow-up of these children [24]. Other studies showed that a fewer clinic visits were associated with poor metabolic control and older children had a lower number of visits [28]. The type of insulin regimen has no association with metabolic control; similar conclusions were present in other studies [29–31]. The type and amount of carbohydrates determine the postprandial glucose [32], dietary compliance in this study is reflected by counting carbohydrates, and hence, the determination of the appropriate dose of insulin is significantly associated with better metabolic control. Counting carbohydrates improved glycemic control which may be linked to more insulin injections, in addition to giving the appropriately needed insulin dose [33, 34]. Consuming carbohydrates at school without insulin is a predictor of poor glycemic control; this finding is expected since only 7.3% of the participants receive insulin injections at school. This raises the issue of optimal management of diabetes at school and overcoming different barriers; limited support at school would deprive children from the needed insulin injections at recess time [35]. Younger children who cannot manage their diabetes effectively need more support at school than older children and adolescents, who can manage their diabetes [36]. An important predictor of metabolic control is the caregiver who decides the insulin doses. Compared to mothers, other family members are not as successful in achieving good metabolic control since HbA1c increased by 0.736%. There is no significant difference in HbA1c between children who determined their own insulin dose and those whose insulin doses were determined by their mothers. Several studies have shown that a mother's knowledge and education played an important role in glycemic control and mothers with more knowledge of diabetes and better education maintained better glycemic control for their children [37]. This is because mothers are usually the primary caregivers conducting coordination and execution of the child's diabetes care plan with the medical team [38].

Contrary to what we expected, there is no significant correlation between the increased frequency of self-monitoring blood glucose (SMBG) and optimal metabolic control, a clear difference from previous reports [14, 39]. The retrospective nature of this study may have contributed to the lack of a statistical significance (*P* value was marginal: 0.052), and hence, we hypothesize that there would be a significant correlation in a future prospective study with a larger sample size. We anticipate that poor compliance for frequent testing of blood glucose is influenced by partial insurance coverage of blood glucose test strips in Jordan and continuous blood glucose sensors are not covered, at all. Other causes for infrequent testing may be behavioral including negligence of testing blood glucose.

To our knowledge, this is the first study to evaluate possible predictors of metabolic control in children with type 1 diabetes in Jordan and is probably among the few studies done in Middle East. It is important to study these factors in developing countries where resources are limited. The fact that there is a universal challenge in achieving optimal metabolic control, in addition to the comparable glycemic control achieved in our Jordanian cohort that resembles one of developing countries with limited health resources [21, 22], delineates the need for global cooperation in setting universal guidelines and developing more effective multidisciplinary diabetes care teams' strategies and better diabetes care education with individualized approach for high-risk patients. Having a registry in each country would help future governmental policies in allocating the proper financial resources on the basis of cost-effective approaches that prevent the cost-prohibitive care ascribed to microvascular and macrovascular diabetes complications.

The limitations of the study include the small sample size and the fact that this study was conducted in two centers in the capital city Amman, both providing superior supervision by medical teams that consist of specialized physicians, dieticians, and registered nurses. Although these centers serve patients referred from different parts of Jordan, more comprehensive prospective studies involving different geographical areas are needed to provide a precise insight into possible predictors and different associations between personal characteristics and metabolic control.

## 5. Conclusion

This study shows that an increase in age of children with type 1 diabetes is associated with deterioration of metabolic control. Dietary compliance and receiving insulin at school lead to reduced HbA1c levels. More attention must be paid to educate mothers and children on counting carbohydrates and to provide support for diabetic children at schools to help them better adhere to their insulin requirements. Mothers are more capable of deciding appropriate insulin doses than other family members. Children who cannot manage their diabetes effectively on their own and have to get the help from family members (other than their mothers) must be paid more attention. Involving these family members in the care plan with appropriate education and knowledge until the child can reach an age where he/she can manage diabetes effectively is essential.

## Figures and Tables

**Table 1:** General characteristics of participants (*N* = 263).

Variables	*N* (%)	Variables	*N* (%)
*Gender (male)*	145 (55.1%)	*Current HbA1c*	
*Duration of diabetes*		<7.5%	55 (20.9%)
≤5 years	218 (82.9%)	7.5%–9.0%	101 (38.4%)
>5 years	45 (17.1%)	>9.0%	107 (40.7%)
*Current age groups (years)*		*Counts of carbohydrates*	
≤5	19 (7.2%)	Yes	88 (33.5%)
>5-10	88 (33.5%)	No	175 (66.5%)
>10-15	117 (44.5%)	*Who decides insulin dose*	
>15	39 (14.8%)	Mother	225 (85.6%)
*First insulin regimen*		Father	28 (10.6%)
MDI	86 (32.7%)	Patient himself/herself	8 (3%)
TDI	177 (67.3%)	Sibling	1 (0.4%)
*DKA as first presentation*		Grandmother	1 (0.4%)
Yes	102 (38.8%)	*Receive insulin at school*	
No	161 (61.2%)	Yes	206 (78.3%)
*Current insulin regimen*		No	26 (9.9%)
MDI	177 (67.3%)	Do not attend school	31 (11.8%)
TDI	84 (31.9%)	*Age at diagnosis (years)*	
Pump	2 (0.8%)	<6	102 (38.8%)
*Upgraded insulin regimen*		>6-14	156 (59.3%)
Yes	96 (36.5%)	>14	5 (1.9%)
No	167 (63.5%)		
*Other comorbidities*			
Celiac	26 (9.9%)		
Hypothyroidism	6 (2.3%)		
Celiac and hypothyroidism	1 (0.4%)		
Others	5 (1.9%)		
None	225 (85.6%)		

**Table 2:** Differences in HbA1c levels among categories of different personal characteristics.

Variables	Mean HbA1c	*P* values	Variables	Mean HbA1c	*P* values
*Gender*		0.817	*Receive insulin at school*		0.007
Female	8.72%		Yes	8.89%	
Male	8.76%		No	8.03%	
*Current age groups (years)*		0.001	Do not attend school	8.38%	
≤5	8.33%		*Other comorbidities*		0.582
>5-10	8.29%		Celiac	8.82%	
>10-15	9.00%		Hypothyroidism	7.74%	
>15	9.19%		Celiac and hypothyroidism	8.80%	
*DKA as first presentation*		0.365	Others	8.81%	
Yes	8.85%		None	8.76%	
No	8.68%		*Who decides insulin dose*		0.006
*First insulin regimen at diagnosis*		0.002	Mother	8.61%	
MDI^Σ^	8.34%		Patient himself/herself	9.28%	
TID^x^	8.94%		Others	9.37%	
*Current insulin regimen*		0.432	*Number of daily glucose testing in last year*		0.052
TID^x^	8.79%		≤4	8.82%	
MDI^Σ^	8.74%		>4	8.34%	
Pump	7.43%		*Count of carbohydrates*		0.004
*Age at diagnosis (years)*		0.145	Yes	8.38%	
≤6	8.53%		No	8.93%	
>6-14	8.87%				
>14	9.26%				
*School grades*		0.002			
<80	9.48%				
80-90	9.04%				
90-100	8.45%				
None^∞^	8.48%				

^Σ^Multiple dose insulin regimen. ^x^Triple dose insulin regimen. ^∞^56% of these children were below school age (1-4.9 years). The rest were in the age group 5-9.9 years.

**Table 3:** Linear regression analysis for possible predictors of HbA1c levels.

Variables	Univariate linear regression			Multivariate linear regression^¥^		
	*B* ^◊^	95% CI	*P* value	*B* ^◊^	95% CI	*P* value
*Age*	0.097	0.049-0.145	<0.001	0.065	0.014-0.116	0.013
*Duration of diabetes*	0.095	0.020-0.170	0.013			
*BMI*	0.073	0.022-0.124	0.005			
*Gender*						
Male^Ø^						
Female	-0.043^∆^	-0.0405-0.320	0.817			
*DKA as presentation*						
Yes^Ø^						
No	-0.170^∆^	-0.0540-0.199	0.36			
*Counts of carbohydrates*						
No^Ø^						
Yes	-0.549	-0.925 to -0.173	0.004	-0.503	-0.870 to -0.135	0.008
*Number of clinic visits during last year of follow-up*						
≤4^Ø^						
>4	0.328	-0.034–0.690	0.075	0.381	0.039–0.723	0.029
*School grades*			0.002			
<80^Ø^						
80-90	-0.439^∆^	-1.116–0.238	0.203			
90-100	-1.034^∆^	-1.697 to -0.371	0.002	-0.353^∆^	-0.698 to -0.009	0.044
None	-1.002^∆^	-1.782 to -0.222	0.012			
*Receive insulin at school*			0.007			
Yes^Ø^						
No	-0.855^∆^	-1.453 to -0.240	0.005	-0.743^∆^	-1.310 to -0.176	0.010
Children who do not attend school	-0.505^∆^	-1.059-0.049	0.074			
*Who decided insulin dose*			0.006			
Mother^Ø^						
Patient himself/herself	0.675	-0.360-1.709	0.200			
Others	0.760	0.271-1.250	0.002	0.580	0.075-1.085	0.024
*Current insulin regimen*			0.432			
TID^x,Ø^						
MDI^Σ^	-0.057	-0.444-0.330	0.772			
Pump	-1.369	-3.458-0.720	0.198			

^**¥**^The model had a *P* value < 0.001, *F* = 6.765, and *R*^2^ = 0.157. ^x^Triple dose insulin regimen. ^Σ^Multiple dose insulin regimen. ^**◊**^Unstandardized coefficient. ^Ø^Reference group. ^∆^Negative values of *B* coefficients represent values of HbA1c lower than those of the reference group.

## Data Availability

The data used to support the findings of this study are included within the article

## References

[1] White N. H., Sun W., Cleary P. A. (2010). Effect of prior intensive therapy in type 1 diabetes on 10-year progression of retinopathy in the DCCT/EDIC: comparison of adults and adolescents. *Diabetes*.

[2] Rewers M. J., Pillay K., de Beaufort C. (2014). ISPAD Clinical Practice Consensus Guidelines 2014. Assessment and monitoring of glycemic control in children and adolescents with diabetes. *Pediatric Diabetes*.

[3] Brink S. J., Chiarelli F. G. (2004). Education and multidisciplinary team approach in childhood diabetes. *Acta Bio-Medica de L'Ateneo Parmense*.

[4] Chiarelli F., Verrotti A., di Ricco L., de Martino M., Morgese G. (1998). Approaches to quality of control in diabetes care. *Hormone Research*.

[5] Nordly S., Jorgensen T., Andreasen A. H., Hermann N., Mortensen H. B. (2003). Quality of diabetes management in children and adolescents in Denmark. *Diabetic Medicine*.

[6] Mohammad H. A., Farghaly H. S., Metwalley K. A., Monazea E. M., Abd El-Hafeez H. A. (2012). Predictors of glycemic control in children with type 1 diabetes mellitus in Assiut-Egypt. *Indian Journal of Endocrinology and Metabolism*.

[7] Spaic T., Mahon J. L., Thind A., Harris S. B. (2013). Predictors of glycemic control in children and adolescents with type 1 diabetes in Southwestern Ontario. *Canadian Journal of Diabetes*.

[8] Goran M. I., Gower B. A. (2001). Longitudinal study on pubertal insulin resistance. *Diabetes*.

[9] Kim H., Elmi A., Henderson C. L., Cogen F. R., Kaplowitz P. B. (2012). Characteristics of children with type 1 diabetes and persistent suboptimal glycemic control. *Journal of Clinical Research in Pediatric Endocrinology*.

[10] Clements M. A., Lind M., Raman S. (2014). Age at diagnosis predicts deterioration in glycaemic control among children and adolescents with type 1 diabetes. *BMJ Open Diabetes Research & Care*.

[11] Secrest A. M., Costacou T., Gutelius B., Miller R. G., Songer T. J., Orchard T. J. (2011). Associations between socioeconomic status and major complications in type 1 diabetes: the Pittsburgh epidemiology of diabetes complication (EDC) study. *Annals of Epidemiology*.

[12] Juarez D. T., Sentell T., Tokumaru S., Goo R., Davis J. W., Mau M. M. (2012). Factors associated with poor glycemic control or wide glycemic variability among diabetes patients in Hawaii, 2006-2009. *Preventing Chronic Disease*.

[13] Cardwell C. R., Patterson C. C., Allen M., Carson D. J. (2005). Diabetes care provision and glycaemic control in Northern Ireland: a UK regional audit. *Archives of Disease in Childhood*.

[14] Miller K. M., Beck R. W., Bergenstal R. M. (2013). Evidence of a strong association between frequency of self-monitoring of blood glucose and hemoglobin A1c levels in T1D exchange clinic registry participants. *Diabetes Care*.

[15] Ababio G. K., Bosomprah S., Olumide A. (2017). Predictors of quality of life in patients with diabetes mellitus in two tertiary health institutions in Ghana and Nigeria. *The Nigerian Postgraduate Medical Journal*.

[16] Al-Agha A. E., Alafif M. M., Abd-Elhameed I. A. (2015). Glycemic control, complications, and associated autoimmune diseases in children and adolescents with type 1 diabetes in Jeddah, Saudi Arabia. *Saudi Medical Journal*.

[17] Niba L. L., Aulinger B., Mbacham W. F., Parhofer K. G. (2017). Predictors of glucose control in children and adolescents with type 1 diabetes: results of a cross-sectional study in Cameroon. *BMC Research Notes*.

[18] Robert A. A., Al-Dawish A., Mujammami M., Dawish M. A. A. (2018). Type 1 diabetes mellitus in Saudi Arabia: a soaring epidemic. *International Journal of Pediatrics*.

[19] Rosenbauer J., Dost A., Karges B. (2012). Improved metabolic control in children and adolescents with type 1 diabetes: a trend analysis using prospective multicenter data from Germany and Austria. *Diabetes Care*.

[20] Damla G. S., Zehra A., Samim Ö. (2013). Diabetes care, glycemic control, complications, and concomitant autoimmune diseases in children with type 1 diabetes in Turkey: a multicenter study. *Journal of Clinical Research in Pediatric Endocrinology*.

[21] Gesuita R., Skrami E., Bonfanti R. (2017). The role of socio-economic and clinical factors on HbA1c in children and adolescents with type 1 diabetes: an Italian multicentre survey. *Pediatric Diabetes*.

[22] Clements S. A., Anger M. D., Bishop F. K. (2013). Lower A1c among adolescents with lower perceived A1c goal: a cross sectional survey. *International Journal of Pediatric Endocrinology*.

[23] Neylon O. M., O'Connell M. A., Skinner T. C., Cameron F. J. (2013). Demographic and personal factors associated with metabolic control and self-care in youth with type 1 diabetes: a systematic review. *Diabetes/Metabolism Research and Reviews*.

[24] Urbach S. L., LaFranchi S., Lambert L., Lapidus J. A., Daneman D., Becker T. M. (2005). Predictors of glucose control in children and adolescents with type 1 diabetes mellitus. *Pediatric Diabetes*.

[25] Hanberger L., Samuelsson U., Lindblad B., Ludvigsson J. (2008). A1C in children and adolescents with diabetes in relation to certain clinical parameters: the Swedish Childhood Diabetes Registry SWEDIABKIDS. *Diabetes Care*.

[26] McCarthy A. M., Lindgren S., Mengeling M. A., Tsalikian E., Engvall J. (2003). Factors associated with academic achievement in children with type 1 diabetes. *Diabetes Care*.

[27] Gonder-Frederick L. A., Zrebiec J. F., Bauchowitz A. U. (2009). Cognitive function is disrupted by both hypo- and hyperglycemia in school-aged children with type 1 diabetes: a field study. *Diabetes Care*.

[28] Kaufman F. R., Halvorson M., Carpenter S. (1999). Association between diabetes control and visits to a multidisciplinary pediatric diabetes clinic. *Pediatrics*.

[29] Holl R. W., Swift P. G., Mortensen H. B. (2003). Insulin injection regimens and metabolic control in an international survey of adolescents with type 1 diabetes over 3 years: results from the Hvidore study group. *European Journal of Pediatrics*.

[30] Mortensen H. B., Villumsen J., Volund A., Petersen K. E., Nerup J., The Danish Study Group of Diabetes in Childhood (1992). Relationship between insulin injection regimen and metabolic control in young Danish type 1 diabetic patients. *Diabetic Medicine*.

[31] Urakami T., Suzuki J., Yoshida A. (2010). Association between sex, age, insulin regimens and glycemic control in children and adolescents with type 1 diabetes. *Journal of Clinical Research in Pediatric Endocrinology*.

[32] Sheard N. F., Clark N. G., Brand-Miller J. C. (2004). Dietary carbohydrate (amount and type) in the prevention and management of diabetes: a statement by the american diabetes association. *Diabetes Care*.

[33] Gökşen D., Atik Altınok Y., Özen S., Demir G., Darcan Ş. (2014). Effects of carbohydrate counting method on metabolic control in children with type 1 diabetes mellitus. *Journal of Clinical Research in Pediatric Endocrinology*.

[34] Dias V. M., Pandini J. A., Nunes R. R. (2010). Effect of the carbohydrate counting method on glycemic control in patients with type 1 diabetes. *Diabetology and Metabolic Syndrome*.

[35] Lawrence S. E., Cummings E. A., Pacaud D., Lynk A., Metzger D. L. (2015). Managing type 1 diabetes in school: recommendations for policy and practice. *Paediatrics & Child Health*.

[36] Nabors L., Lehmkuhl H., Christos N., Andreone T. L. (2003). Children with diabetes: perceptions of supports for self-management at school. *The Journal of School Health*.

[37] Al-Odayani A. N., Alsharqi O. Z., Ahmad A. M., Khalaf Ahmad A. M., Al-Borie H. M., Qattan A. M. (2013). Children’s glycemic control: mother’s knowledge and socioeconomic status. *Global Journal of Health Science*.

[38] Pendley J. S., Kasmen L. J., Miller D. L., Donze J., Swenson C., Reeves G. (2002). Peer and family support in children and adolescents with type 1 diabetes. *Journal of Pediatric Psychology*.

[39] Helgeson V. S., Honcharuk E., Becker D., Escobar O., Siminerio L. (2011). A focus on blood glucose monitoring: relation to glycemic control and determinants of frequency. *Pediatric Diabetes*.

